# Mesenchymal stem cell-derived extracellular vesicles in systemic sclerosis: role and therapeutic directions

**DOI:** 10.3389/fcell.2024.1492821

**Published:** 2024-10-17

**Authors:** Xuan Wang, Jiaying Guo, Qiangfu Dai

**Affiliations:** ^1^ Department of Rheumatology and Immunology, The Second Affiliated Hospital of Wannan Medical College, Wuhu, China; ^2^ Department of Geriatric Medicine, The Second Affiliated Hospital of Wannan Medical College, Wuhu, China

**Keywords:** systemic sclerosis, extracellular vesicles, mesenchymal stem cells, fibrosis, autoimmune disease

## Abstract

Systemic sclerosis (SSc) is a complex autoimmune disease with clinical symptoms of vascular damage, immune disorders, and fibrosis, presenting significant treatment challenges and limited therapeutic options. Mesenchymal stem cell-derived extracellular vesicles (MSC-EVs) have been demonstrated in numerous studies as more effective than MSCs in treating autoimmune diseases. Recent studies demonstrate that MSC-EVs can significantly ameliorate the symptoms of SSc and mitigate pathological changes such as vascular injury, immune dysregulation, and fibrosis. These findings underscore the promising therapeutic potential of MSC-EVs in the treatment of SSc. MSC-EVs promote angiogenesis, modulate immune dysfunction, and combat fibrosis. This article summarizes the therapeutic applications and possible mechanisms of MSC-EVs for SSc, thereby offering a novel therapeutic direction for the treatment of SSc.

## 1 Introduction

Systemic sclerosis (SSc) is a rare autoimmune connective tissue disease with an overall prevalence of 17.6 per 1,00,000 individuals. The disease’s comorbidity and prevalence are five times higher in women than in men (Bairkdar et al., 2021). SSc exhibits a complex pathogenesis and highly heterogeneous clinical presentation (Di Battista et al., 2023). The main characteristics of SSc include vasculopathy, immune dysfunction, and fibrosis (Lescoat et al., 2021; Stifano and De Palma, 2021; Yin et al., 2024). Early symptoms of SSc are subtle and easily overlooked, whereas late-stage symptoms are more apparent and easier to diagnose (Rosendahl et al., 2022). Consequently, the disease is often diagnosed at an advanced stage, complicating treatment and increasing mortality rates. The pathogenesis of SSc primarily involves early microvascular changes accompanied by endothelial cell dysfunction, followed by infiltration of lymphocytes and histiocytes around affected vessels. This process results in extracellular matrix deposition and myofibroblast activation (Frech et al., 2022). The early stages of SSc are characterized by Raynaud’s phenomenon, often referred to as “very early SSc” (Bellando-Randone and Matucci-Cerinic, 2019). The hallmark clinical feature of SSc is progressive and extensive fibrosis of the skin and internal organs, accompanied by organ-related complications, which severely impact the patient’s quality of life and psychological wellbeing (Frech et al., 2022; Hughes and Herrick, 2023; Moysidou et al., 2023). SSc is characterized by fibrosis of the lesions and is often accompanied by organ complications. Examples include scleroderma renal crisis (SRC) (Cole and Ong, 2023), SSc-associated interstitial lung disease (Perelas et al., 2020), and cardiac involvement (Moysidou et al., 2023). These complications significantly contribute to the high mortality rate in SSc patients. Currently, there is no single therapeutic approach that is universally effective for all SSc patients. Consequently, existing studies emphasize the need for developing personalized treatments tailored to the disease progression in individual patients, aiming to alleviate various symptoms and improve their quality of life. The use of immunomodulators in SSc patients targets reducing inflammation and fibrosis, while vascular therapeutic agents like sildenafil have shown efficacy in treating pulmonary hypertension (Volkmann et al., 2023).

The etiology of Systemic Sclerosis (SSc) is currently complex and not well understood. Numerous studies have indicated that environmental factors, medications, microbial imbalances, and genetic factors may contribute to the development of SSc (Khanna et al., 2020; Alahmari et la., 2022). Genetic factors are considered the main contributors to the pathogenesis. Different studies show that family members of SSc patients have a significantly higher risk of developing the disease compared to the general population, with a familial aggregation rate as high as 0.72. Furthermore, first-degree relatives of SSc patients have a significantly increased probability of developing other autoimmune diseases (Kuo et al., 2016). The strongest genetic association of SSc has been reported at the major histocompatibility complex (MHC) locus. One study investigated the genetic component of the MHC locus and found that the primary genetic contribution to the disease came from the human leukocyte antigen (HLA) region. Genes such as HLA-DRB111:04, HLA-DQB102:02, and HLA-DPB1*13:01 in HLA class II were strongly associated with genetic susceptibility to SSc. Additionally, for the first time, their study emphasized the involvement of HLA class I genes in the pathogenesis of SSc, an area previously underexplored. These allelic associations may pave the way for precision therapies and could develop into potential molecular biomarkers (Acosta-Herrera et al., 2021; Hanson et al., 2022).

Mesenchymal stem cells (MSCs), first isolated from bone marrow in 1970, are multipotent stem cells with multidirectional differentiation and self-replenishing abilities. MSCs can secrete various cytokines, extracellular vesicles (EVs), inflammatory factors, chemokines, and more to perform functions such as immunomodulation and stimulation of tissue regeneration (Wechsler et al., 2021; Wang et al., 2022). For decades, MSCs have been used as therapeutic agents to replace dead or damaged cells in animal and clinical trials (Harman et al., 2021; Shimizu et al., 2022; Tan et al., 2022). Recent research highlights the significant potential of MSCs in disease treatment through the release of EVs, particularly in conditions such as osteoarthritis, neurological disorders, and renal disorders (Yang et al., 2021; Roth et al., 2022; An et al., 2023). For instance, research demonstrates that human gingival MSCs (hGMSCs) exhibit both pro-angiogenic and anti-inflammatory properties. Moreover, the EVs they secrete offer protection to cardiomyocytes in hypoxic environments (Della Rocca et al., 2023). EVs contain a variety of active substances, including proteins and nucleic acids, which enable them to participate in cellular communication, influence immune and inflammatory regulation, deliver genetic material, and affect receptor cells (Mas-Bargues and Alique, 2023; Aloi et al., 2024). Additionally, EVs have the advantage of crossing the blood-brain barrier, protecting the carried material from enzymatic degradation, and exhibiting low immunogenicity (Chi et al., 2022), which makes MSC-derived EVs promising for SSc therapy. All cells can secrete EVs, which are small lipid bilayer vesicles with diameters ranging from 30 to 2000 nm (Huang et al., 2022). However, current EV isolation techniques do not differentiate between the various types of EVs produced by different mechanisms. The nomenclature for EV subtypes in the guidelines published by the International Society for Extracellular Vesicles (ISEV) in 2014 and 2018 was found to be insufficient, prompting the ISEV to update its guidelines in 2023. The new guidelines discourage the use of biogenesis-based terms such as exosomes and microvesicles. Instead, ISEV recommends using the generic term “EV” and specific extensions as needed. Generally, EVs less than 200 nm in diameter are referred to as small extracellular vesicles (sEVs), while those greater than 200 nm are called large extracellular vesicles (LEVs). ISEV has also introduced new terms for EV mimics: artificial cell-derived vesicles (ACDVs) for those produced by inducing cellular rupture in the laboratory, and synthetic vesicles (SVs) for those synthesized from molecular components (Welsh et al., 2024). In this review, the authors use the terms sEVs or LEVs based on EV size and ACDVs or SVs based on their synthesis method. When the original study does not specify EV size or synthesis method, the term “EVs” is used uniformly.

The application of MSC-derived EVs in clinical settings presents several challenges, such as susceptibility to macrophage clearance (Imai et al., 2015) and difficulties in achieving high and sustained yields (Welsh et al., 2024). However, MSC-derived EVs offer numerous advantages over MSC transplantation in disease treatment, including low immunogenicity, the capability to cross the blood-brain barrier, ease of storage, and modifiability (Zhang et al., 2022; Wang et al., 2024). As a result, numerous studies are currently investigating the application of MSC-derived EVs in the treatment of SSc. This paper provides an overview of the pathogenesis of SSc and the potential applications of MSC-derived EVs in SSc therapy.

## 2 Pathogenesis of systemic sclerosis

**FIGURE 1:**
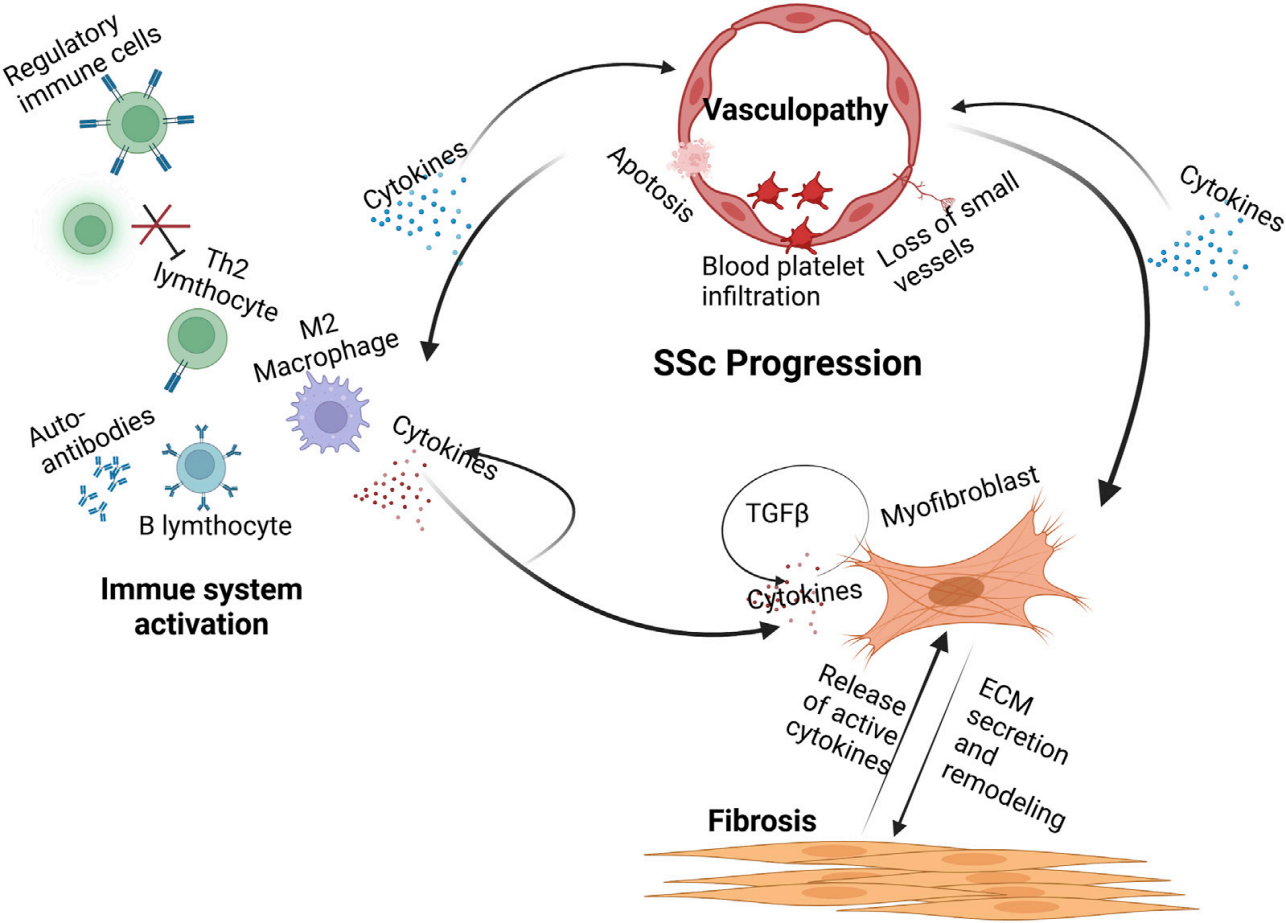
Pathogenesis of systemic sclerosis. Early injury to the vascular system leads to endothelial cell apoptosis, immune cell infiltration, and loss of small vessels. These processes release cytokines that activate cells of the innate and adaptive immune systems. These immune cells release a variety of proinflammatory and fibrogenic cytokines. B cells secrete autoantibodies against nuclear and other antigens and are implicated in tissue injury. Myofibroblasts secrete high levels of ECM components within the affected tissues, leading to severe stiffness and rigidity. (Created with BioRender.com).

The exact etiology of systemic sclerosis (SSc) remains unclear, although numerous studies suggest that both exogenous and endogenous environmental factors may trigger the activation of relevant genes. SSc episodes lead to early vascular injury, followed by activation of the immune system. These factors eventually cause fibroblast fibrosis and extracellular matrix deposition (Figure 1) (Frech et al., 2022). The progression of SSc pathogenesis can be understood through three main aspects: vascular injury, immune system activation, and fibrosis.

### 2.1 Vascular injuries

Vasculopathy is an early change in systemic sclerosis (SSc), initiated by predisposing factors, including infection, oxidative stress, cytotoxicity, and occupational exposure (Maehara et al., 2020, ER, 2022; Kizilay Mancini et al., 2022; Blum et al., 2023). Initially, these factors cause endothelial cell swelling or apoptosis, accompanied by enhanced expression of adhesion molecules like E-selectin (Deng et al., 2021; Romano et al., 2022). This leads to aberrant activation of vasoactive factors. Adhesion molecules recruit inflammatory cells, while disruption of vasoactive factors maintains elevated microvascular tone. Alterations in the microvasculature promote platelet activation, increasing vascular permeability and resulting in microvascular leakage. As a result, vascular smooth muscle cells and pericytes proliferate, leading to thickening of the vessel wall and narrowing of the lumen (Hegner et al., 2016; Rius et al., 2024).

### 2.2 Immune system activation

In the early stages of systemic sclerosis (SSc), immune cell infiltration, including T cells, dendritic cells, macrophages, and monocytes, takes place before detectable endothelial cell damage (Bosello et al., 2018). Pro-inflammatory factors are elevated during these initial stages and correlate with the severity of SSc. The relationship between vascular injury and the immune response is intimately linked and interdependent.

Immune cells secrete various fibrotic factors, including IL-4, IL-13 (Gasparini et al., 2020), IL-17 (Wei et al., 2022), IL-11 (Steadman and O'Reilly, 2023), IL-6 (Lin et al., 2022), tumor necrosis factor-α (TNF-α) (Kosałka-Węgiel et al., 2024), and transforming growth factor-β (TGF-β) (Lomelí-Nieto et al., 2023), and these factors are significantly elevated in the serum of patients with SSc (Zarrabi et al., 2021; Kosałka-Węgiel et al., 2024).

Abnormal activation of macrophages may contribute to chronic inflammation and fibrosis. Thus, the role of macrophages in modulating inflammation and fibrosis in SSc is gradually being recognized. Macrophages can be stimulated to differentiate into two primary phenotypes: classically activated M1-like macrophages and alternatively activated M2-like macrophages (Boutilier and Elsawa, 2021). These macrophage phenotypes are primarily distinguished by their surface markers (Wang et al., 2024). During the early stages of SSc, M1-like macrophages are significantly increased, secreting pro-inflammatory factors such as TNF-α and IL-6 (Wang et al., 2022; Peng et al., 2023). M2-like macrophages are activated during the late repair phase of the disease and inhibit the M1 response by releasing anti-inflammatory factors such as IL-4 and IL-13. Additionally, they promote the release of extracellular matrix (ECM) proteins and pro-fibrotic factors, and trigger Th2 effectors to enhance anti-inflammatory responses (Hu et al., 2023). Therefore, M2-like macrophages are considered a crucial pathogenic factor in SSc.

T cells play a crucial role in the pathogenesis of systemic sclerosis (SSc) as part of the adaptive immune response. CD4^+^ and CD8^+^ T cells are present in the skin and lungs of patients with SSc(Servaas et al., 2021; Frantz et al., 2022; Yang et al., 2022). CD4^+^ T cells differentiate into helper T (Th)1, Th2, Th17, and regulatory T (Treg) cells (Jin et al., 2022). The Th1/Th2 balance is crucial in the regulation of allergies and autoimmune diseases. Studies have shown that in patients with SSc, the Th2/Th1 ratio is significantly elevated, with Th2 cells playing a more dominant role in the pathogenesis of SSc compared to the interferon-γ (IFN-γ) produced by Th1 cells. Th2 cells produce pro-fibrotic factors, such as IL-4 and IL-13, which stimulate fibroblasts to synthesize collagen, thereby advancing the fibrotic process. Therefore, targeting Th2 cell differentiation represents a potential therapeutic strategy for SSc (Tian et al., 2024). IL-17, secreted by Th17 cells, is significantly elevated in patients with advanced SSc and is positively correlated with disease severity (Han et al., 2022). Treg cells are significantly elevated in patients with SSc; however, their impaired immunosuppressive function suggests that targeting Treg cells for depletion could be a novel therapeutic approach for SSc (Liu et al., 2021).

Patients with SSc also exhibit an imbalance in B-cell homeostasis in their blood. An increased number of activated naïve B cells and a decrease in memory B cells are observed. The B-cell activating factor (BAFF) plays a crucial role in B-cell proliferation and maturation, and its elevated levels may directly contribute to the pathogenesis of SSc. Studies have shown that patients with SSc exhibit elevated serum levels of anti-BAFF autoantibodies (Erdő-Bonyár et al., 2022).

### 2.3 Fibrosis

Persistent injury, inflammation, and immune cell activation lead to fibroblast activation, contributing to the progression of SSc towards fibrosis. The clinicopathologic hallmarks of fibrosis include the proliferation of myofibroblasts and the accumulation of extracellular matrix (ECM) proteins within tissues, leading to organ damage and eventual failure (Abraham et al., 2024). Numerous studies have demonstrated that myofibroblast transformation is primarily induced by epithelial cells via epithelial-mesenchymal transition (EMT). Beyond its pro-fibrotic role in various autoimmune diseases, EMT is also critical to the fibrotic processes in tumors and chronic fibrotic diseases (e.g., diabetic nephropathy, end-stage liver disease) (Li et al., 2022; Du et al., 2023; Zhou et al., 2023a). The chronic inflammatory state in the pathogenesis of SSc serves as an effective trigger for EMT, promoting the development of pathological fibrosis. Epithelial cells are primarily connected through various types of outer cell-cell junctions, which transition to a spindle shape upon dissolution of these junctions. Once EMT is initiated, epithelial cells undergo a transition to a fully mesenchymal state, characterized by the loss of epithelial markers such as E-cadherin and the upregulation of mesenchymal markers like fibronectin, vimentin, and N-cadherin (Utsunomiya et al., 2022; Sarrand and MS, 2023).

Transforming growth factor-beta (TGFβ) is a central mediator of pathological fibrosis and is pivotal in numerous biological processes, including wound repair, cell differentiation, and immune regulation (Sun et al., 2024). Beyond its involvement in autoimmune diseases, TGFβ also plays a critical role in conditions such as tumors and diabetic nephropathy. In tumor pathogenesis, TGFβ activation directly induces EMT in tumor cells, leading to the deposition of ECM proteins (Canè et al., 2021). TGFβ1 has been identified as a diagnostic marker for skin and lung fibrosis in SSc (Martinović Kaliterna and Petrić, 2019). Additionally, TGFβ activates connective tissue growth factor (CTGF), which synergistically regulates fibrosis alongside TGFβ (Nakai et al., 2019). Other molecules that exert synergistic effects with TGFβ in fibrosis include platelet-derived growth factor (PDGF). CXCL4 is a chemokine produced by platelets and various immune cells under pathological conditions, with elevated levels detected in the skin and circulation of SSc patients. A study demonstrated that stimulation of monocytes from SSc patients with CXCL4 induces the release of PDGF-BB, thereby exacerbating fibrosis (van der Kroef et al., 2020).

In summary, the initial injury and chronic inflammation in SSc trigger the production of TGFβ, CTGF, PDGF, among other factors, which in turn initiate the EMT process. This facilitates the differentiation of more cells into myofibroblasts, thereby exacerbating fibrosis (Dees et al., 2020).

## 3 MSC-EVs in the treatment of SSc

**FIGURE 2:**
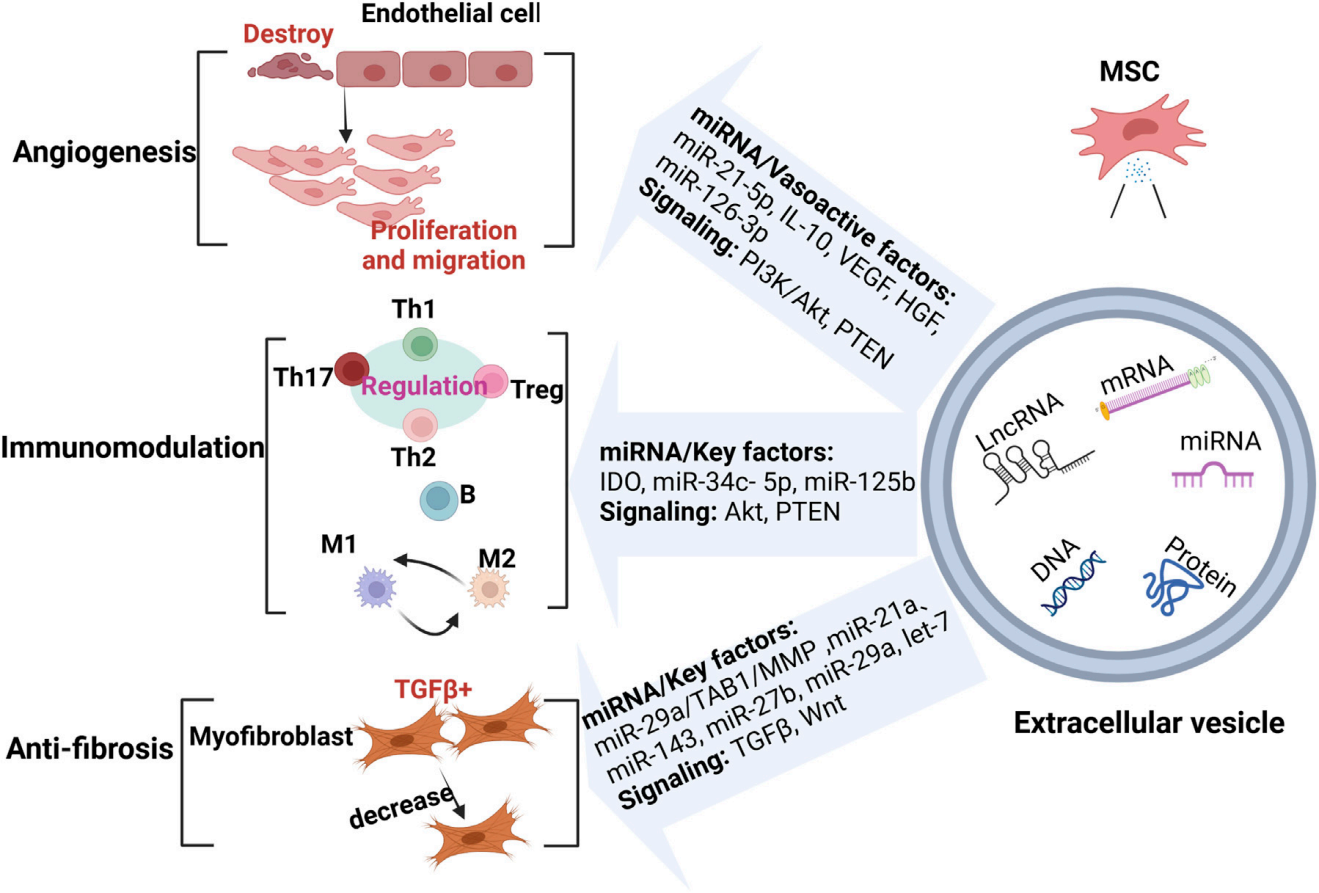
MSC-EVs in the treatment of SSc. MSC-EVs exert therapeutic effects by enhancing angiogenesis, modulating the immune response, and reducing fibrosis through miRNAs and key molecules. (Created with BioRender.com).

MSC-EVs represent a novel therapeutic approach, emerging as an innovative modality in tumor therapy, either as a drug carrier or as a tumor vaccine (Huang et al., 2022; Wang et al., 2022a). Current therapeutic approaches for SSc primarily include anti-vascular injury, anti-fibrotic, and immunomodulatory therapies. Common therapeutic agents include: (a) Nidanib, which exhibits antifibrotic effects and has proven effective in SSc patients with interstitial lung disease, slowing the progression of pulmonary fibrosis; dabigatran, a direct thrombin inhibitor, has shown efficacy in improving dermatosclerosis symptoms in small-scale clinical trials (Kowal-Bielecka et al., 2017; Campochiaro and Allanore, 2021; Teske and Fett, 2024). (b) Leflunomide, used to treat SSc patients with skin and lung involvement, is strongly supported by recent EULAR recommendations; cyclophosphamide remains a key immunosuppressant before stem cell transplantation in critically ill patients (Teske and Fett, 2024). (c) Phosphodiesterase 5 inhibitors and endothelin receptor antagonists are commonly used to treat pulmonary hypertension and Raynaud’s phenomenon, playing a crucial role in mitigating early vascular damage (Distle et al., 2024). Although these therapies can effectively control the symptoms of SSc, they cannot cure the disease. Recent exploration of MSC-EVs as a treatment for SSc represents a significant breakthrough in the field. Given that MSC-EVs are enriched with various potent biological factors, their documented successes in combating vascular injury, fibrosis, and immunomodulation in SSc suggest they may become a valuable tool for SSc treatment (Figure 2) (Rozier et al., 2021a), as detailed below.

### 3.1 Angiogenesis

Vascular injury is a key part of SSc pathogenesis and occurs at an early stage; therefore, timely treatment and control during this stage can greatly reduce fibrosis and complications in the later stages of the disease (Kowal-Bielecka et al., 2009). MSC-EVs exert their therapeutic functions through the delivery of genetic material, including cytokines, miRNAs, and LncRNAs, which they transport to the relevant receptor cells. They are particularly effective in wound healing and ischemic disorders, such as diabetic foot ulcers and myocardial infarction, with strong pro-angiogenic effects (Song et al., 2021; Kouroupis et al., 2023). A recent study developed a novel hydrogel dressing, HMN/TP, designed for rapid wound protection, particularly in resource-limited settings. The hydrogel also exhibits anti-inflammatory and pro-vascular regeneration properties (Li et al., 2024). This research strongly suggests that MSC-EVs can be engineered to enhance and stabilize their pro-vascular effects across various diseases.

MSC-EVs are rich in various anti-inflammatory and antioxidant molecules that mitigate SSc-induced inflammation and oxidative stress through multiple pathways, thereby protecting vascular endothelial cells and reducing vascular damage. IL-10, present in MSC-EVs, is a potent anti-inflammatory factor that inhibits the release of pro-inflammatory cytokines, including TNF-α, IL-6, and IL-1β. This action reduces the inflammatory response in vascular endothelial cells and helps preserve the vascular integrity (Jiang et al., 2022). MSC-EVs are also enriched in Hepatocyte Growth Factor (HGF) and Vascular Endothelial Growth Factor (VEGF). HGF promotes the proliferation and migration of endothelial cells (Wang et al., 2017; Chen et al., 2019), while VEGF facilitates the repair of damaged blood vessels by promoting angiogenesis, thereby restoring normal vascular function (Phelps et al., 2023).

MSCs have the potential for multidirectional differentiation and are primarily derived from the umbilical cord (UC), bone marrow (BM), and adipose tissue (AD). MSCs localize to the site of injury and secrete anti-inflammatory molecules and growth factors through paracrine, autocrine, and endocrine mechanisms to accelerate wound healing (Hoang et al., 2020; Lu et al., 2022). MSC-derived EVs from all these sources are crucial for vascular injury repair. For example, AD-derived MSC-sEVs can enhance the migration and proliferation of endothelial cells (ECs), thereby facilitating wound healing in diabetic nephropathy (Song et al., 2023). UC-derived MSC-sEVs have also been shown to facilitate wound healing in diabetic conditions (Yang et al., 2020). miRNAs in MSC-sEVs can promote neovascularization by modulating gene expression, thereby promoting endothelial cell proliferation and migration. Zhu et al. found that miR-126-3p carried by sEVs can be internalized by human umbilical vein endothelial cells (HUVECs), promoting their proliferation and tube formation via the mTOR pathway, thereby enhancing angiogenesis (Zhu et al., 2024). MSC-EVs are inherently angiogenic. Recent studies have shown that the angiogenic potential of sEVs can be enhanced by engineering adipose tissue-derived stem cells (ADSCs) to produce ADSCs-ACDVs, which are enriched in miR-21-5p. Co-incubation of these ADSCs-ACDVs with HUVECs led to a marked reduction in PTEN protein levels and a notable increase in VEGF levels in HUVECs, indicating that ADSCs-ACDVs promote angiogenesis through PTEN inhibition and activation of the PI3K/Akt pathway (Sun et al., 2022). Therefore, autologous MSC-ECDVs, which often have low yield and purity, could be more efficiently employed for the treatment of vascular injury through engineering approaches.

### 3.2 Immunomodulation

In SSc, the pathological activation primarily includes dysregulation of Th1, Th2, Th17, Treg cells, and M1/M2 cytokines (Qin et al., 2023; Yang et al., 2023a). MSC-EVs function as immunomodulators in SSc by modulating the production and differentiation of various immune cells, including T cells, B cells, and macrophages (Zhao et al., 2023; Zhao et al., 2023a).

#### 3.2.1 T cells

Suppression of inflammation is also a crucial therapeutic approach in the early stages of SSc pathogenesis. MSC-EVs can activate different effectors in response to distinct microenvironments to modulate immune disorders in SSc.

MSC-sEVs can modulate the immune response through modulating the balance between Th17 and Treg cells. Th17 cells secrete IL-17, which exerts a pro-inflammatory effect, while Tregs exert an immunosuppressive function by secreting anti-inflammatory factors such as IL-10, TGF-β, and IL-35 (Hu et al., 2024). Jung et al. demonstrated that MSC-sEVs can be internalized by naïve CD4^+^ T cells, thereby modulating their differentiation and preventing the conversion of CD4^+^ T cells into Th17 cells. RAR-associated orphan receptor γt (RORγt) is the key transcription factor for defining the Th17 lineage, and its stability is maintained by acetylation and K63-linked polyubiquitination. Their study revealed that MSC-sEVs induce the degradation of RORγt through K63-linked deubiquitination, leading to reduced IL-17 production in CD4^+^ T cells polarized to Th17 cells. This mechanism exerts anti-inflammatory and immunosuppressive effects in autoimmune diseases (Jung et al., 2023). Conversely, MSCs induce FoxP3 expression through the secretion of indoleamine 2,3-dioxygenase (IDO), which increases the proportion of Tregs in the spleen of EAE patients, thereby alleviating clinical symptoms and disease severity in EAE (Manganeli Polonio et al., 2021).

Maintaining the Th1/Th2 balance is crucial in the pathogenesis of autoimmune diseases. Yang et al. (2023) showed that ADSCs-sEVs can inhibit Th2 differentiation and reduce the serum levels of sIgE, IL-4, and IFN-γ, which are key markers of allergic rhinitis (AR), thereby significantly improving the allergic symptoms of AR, such as sneezing.

#### 3.2.2 Macrophages

Macrophage polarization is crucial for regulating the tumor microenvironment, and dysregulation of the M1/M2 ratio plays a key role in the inflammatory response and fibrosis associated with autoimmune diseases (Yang et al., 2023). AKT is a pivotal protein in M1 polarization; its phosphorylation promotes M1 polarization while inhibiting M2 polarization. Diabetic wound healing is frequently delayed by inflammatory factors, which can ultimately result in amputation. Bone marrow mesenchymal stem cell (BMSC)-derived sEVs significantly upregulated PTEN expression, inhibiting AKT phosphorylation and thereby shifting the balance toward M2 polarization. Additionally, MSC-sEVs can enhance collagen synthesis and angiogenesis, thereby promoting wound healing (Liu et al., 2020). Hu et al. (2022) demonstrated that MSC-sEVs may transfer miR-34c-5p to macrophages via a CD81-epidermal growth factor receptor (EGFR) complex, thereby suppressing macrophage activation.

#### 3.2.3 B cells

B lymphocytes are crucial in the adaptive immune response, and MSC-EVs have been shown to regulate both the proliferation and function of B cells. Xing et al. isolated labial gland (LG)-derived MSC sEVs (LGMSCs-sEVs) and found that LGMSCs-sEVs could attenuate the symptoms of primary Sjögren’s syndrome (pSS) by regulating B cell proliferation and differentiation. pSS results from abnormalities or over-activation of T and B cells. They injected LGMSCs-sEVs into mice with pSS, resulting in a significant reduction in the proportion of CD19^−^and CD138+ plasma cells in the spleen. Sequencing analysis suggested that miR-125b may be pivotal in mediating the inhibition of plasma cell differentiation (Xing et al., 2022).

### 3.3 Anti-fibrotic effect

Fibrosis in SSc represents a more advanced stage of the disease, characterized not only by skin fibrosis but also by severe fibrosis of vital organs. Research on MSC-EVs has increasingly focused on their role in addressing fibrosis. Reduced levels of miR-29a have been detected in the serum and skin tissues of patients with SSc. miR-29a is an antifibrotic factor, negatively correlated with type I collagen levels. Rozier et al. demonstrated that human AD-derived MSC-EVs release miR-29a, which targets TGFβ-activated kinase 1-binding protein 1 (TAB1), leading to decreased TIMP1 expression and increased matrix metalloproteinase 1 (MMP1) levels, ultimately exerting antifibrotic effects (Rozier et al., 2021b). Additionally, they preactivated MSC-EVs with IFNγ, which effectively improved lung fibrosis in mice; however, a low dose of IFNγ did not significantly impact the reduction of skin thickening (Rozier et al., 2021c). Jin et al. (2021) developed an SSc mouse model by injecting bleomycin (BLM) and subsequently treated the mice with isolated mouse BMSC-derived EVs. In the skin of BLM-treated mice, TGF-β1-positive cells and α-SMA-positive myofibroblasts were significantly increased. However, in the SSc model mice treated with BMSC-EVs, the number of TGF-β1-positive cells was significantly reduced. Subsequently, through sequencing the miRNAs in BMSC-EVs, they identified that miR-21a, miR-143, miR-27b, miR-29a, and let-7 were highly enriched. GO analysis predicted that these miRNAs are associated with classical pathways such as WNT and TGFβ, making them potential therapeutic targets for the disease. Additionally, they found that BMSC-EVs could reduce pro-inflammatory cytokines in the SSc mouse model.

There has been extensive research and application of MSC-EVs in various other diseases. Currently, the use of MSC-EVs in SSc treatment primarily focuses on antifibrotic therapy. Therefore, our research on the angiogenic and immunomodulatory aspects of SSc represents a promising and valuable new avenue to enhance the quality of life for SSc patients.

## 4 Conclusion and outlook

MSC-EVs can ameliorate vascular injury, immune dysfunction, and fibrosis in SSc by modulating key factors and signaling pathways. Numerous studies now indicate a positive role of MSC-EVs in the treatment of SSc. Additionally, some research indicates that pluripotent stem cells derived from hGMSCs reprogramming (iPS), along with iPS-derived EVs, may offer superior therapeutic advantages (Della Rocca et al., 2024). However, further clinical trials are required to confirm these findings. A key challenge that needs to be addressed is scaling up and engineering large-scale MSC-EVs production, which could enhance their therapeutic potency and stability. This includes the genetic modification of MSCs to achieve specific therapeutic outcomes.
